# Artificial intelligence in radiation therapy: from imaging to delivery—a comprehensive review

**DOI:** 10.1093/bjrai/ubaf012

**Published:** 2025-07-11

**Authors:** Kristen Duke, Nikos Papanikolaou

**Affiliations:** Department of Radiation Oncology, The University of Texas Health Science Center at San Antonio, San Antonio, TX 78229, United States; Department of Radiation Oncology, The University of Texas Health Science Center at San Antonio, San Antonio, TX 78229, United States

**Keywords:** artificial intelligence, machine learning, deep learning, radiotherapy, radiation oncology, clinical trials, regulations, radiation therapy, personalized medicine

## Abstract

Artificial intelligence (AI) is revolutionizing radiotherapy by enhancing precision, efficiency, and personalization in patient care. AI offers innovative solutions to longstanding challenges in radiation oncology by streamlining workflows, improving treatment accuracy, and alleviating the burden of heavy workloads on healthcare professionals, which is particularly critical in addressing current staffing shortages. As AI continues to evolve, its applications in radiotherapy are expected to expand, underscoring the need for healthcare providers to understand its current uses and future potential. This review provides a comprehensive analysis of AI’s role across all stages of the radiotherapy workflow, from imaging and treatment planning to quality assurance and delivery. By focusing on recent advancements and key areas of implementation, the review highlights how AI is transforming clinical workflows and improving patient outcomes. Additionally, this review addresses the critical topic of AI regulations and guidelines, which are essential for its safe and ethical integration into clinical practice. It also explores AI’s applications in clinical trials, emphasizing its role in enhancing trial efficiency and reliability. By examining these aspects, this review offers a detailed overview of the current state of AI in radiotherapy and its potential to shape the future of cancer treatment.

## Introduction

### AI’s growing role in radiotherapy

**Figure 1.**
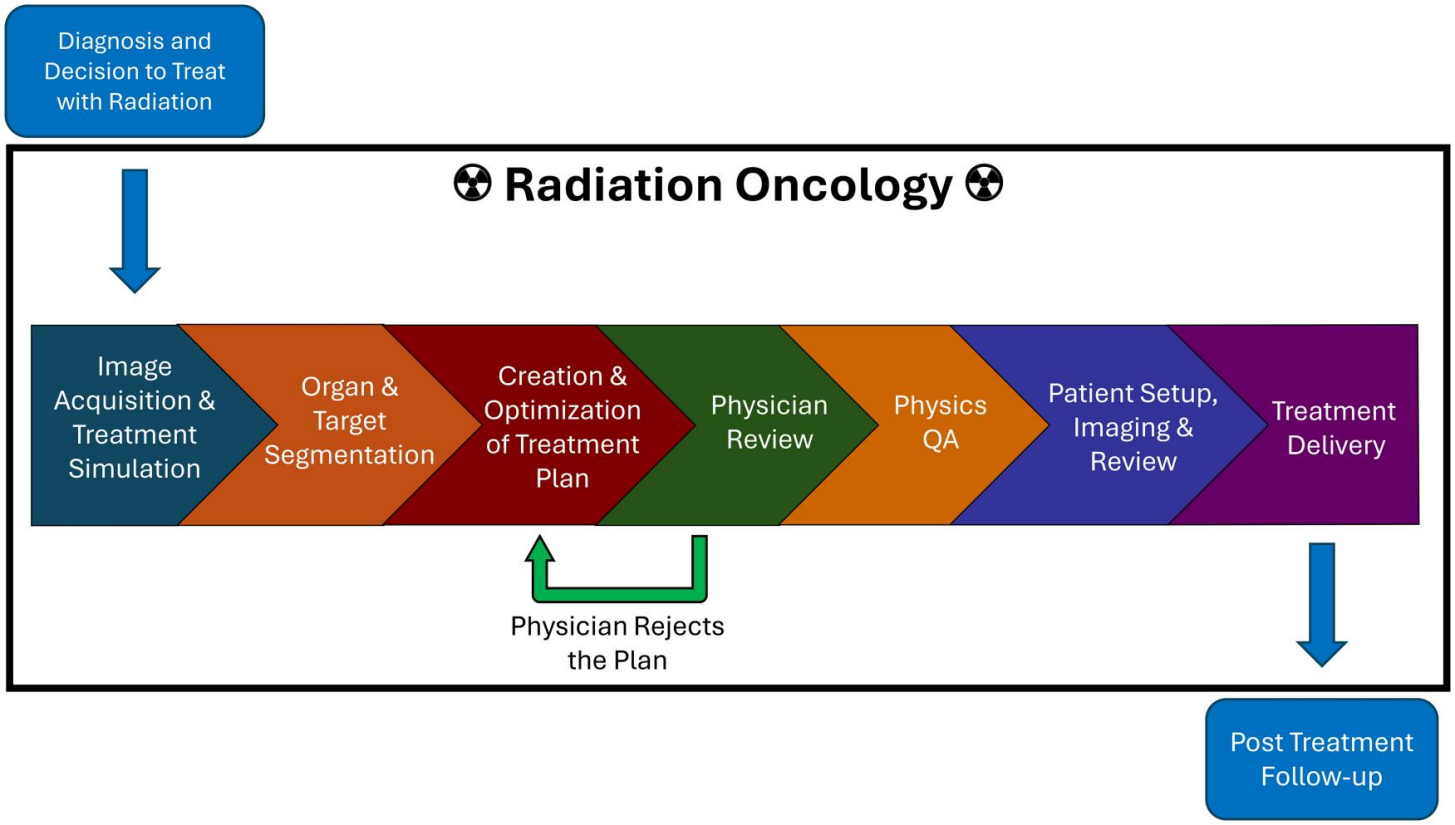
The radiation oncology process.

Artificial intelligence (AI) is a field of computer science focused on creating systems capable of performing complex tasks that typically require human intelligence. AI offers solutions that improve precision, efficiency, and personalization in cancer treatments. AI algorithms are increasingly integrated into clinical workflows, enabling advancements in imaging, treatment planning, quality assurance (QA), and adaptive radiotherapy (ART). These developments aim to address longstanding challenges, including resource optimization and the need for greater accuracy in treatment delivery. While many advancements remain in the research phase, some, like image registration systems and automated contouring, are already in clinical use, demonstrating AI’s transformative potential in patient care. This review examines AI’s contributions at every stage of the radiotherapy process, following the patient’s journey through radiation oncology, as seen in Figure 1.

### Objectives and novelty

Integrating AI into hospitals requires physicians to understand and trust these technologies for patient care, and its importance is heightened by workforce shortages in healthcare. A 2023 American Society for Radiation Oncology survey revealed that 93% of radiation oncologists reported staff shortages, with 53% acknowledging that these shortages are causing treatment delays.^1^ According to the American Association of Physicists in Medicine’s (AAPM) TG-100, the leading cause of treatment errors in intensity-modulated radiation therapy (IMRT) include human factors such as fatigue and stress due to overwork, and inadequate staffing levels.^2^ These factors highlight AI’s pivotal role in overcoming challenges by streamlining workflows, minimizing errors, and improving treatment precision.

This paper comprehensively analyses AI’s integration across the radiotherapy workflow while exploring emerging topics such as regulatory frameworks and clinical trial use. These areas, which have become a focal point for advancing AI adoption, are essential for ensuring its safe and effective implementation in clinical practice. Additionally, while a detailed explanation of AI architectures or methodologies is beyond the scope of this review, readers seeking foundational information on the basics of AI in healthcare can refer to Meskó and Görög.^3^ For a deeper understanding of AI architectures and techniques, Sarker’s comprehensive review provides valuable insight into advanced methodologies.^4^

## Imaging and simulation

The imaging and simulation phase marks the first step of a patient’s journey in radiotherapy. AI advancements are enhancing the efficiency and accuracy of image processes by improving image quality for treatment planning and enabling dose reductions. Key applications include image reconstruction specifically for radiotherapy, synthetic data generation, and image registration.

### Imaging reconstruction

AI methodologies are advancing medical image reconstruction within radiotherapy workflows. Shan et al introduced a deep learning (DL) network to reconstruct distortion-free images from inhomogeneity-corrupted data for MRI-guided radiotherapy (MRgRT), enabling accurate and rapid guidance.^5^ Yasui et al validated a DL-based reconstruction approach for planning CT images, showing improved image quality and reliable electron density estimations across varied dose settings, enabling potential dose reduction without sacrificing accuracy.^6^ These innovations provide clinicians with higher-quality images for more precise treatment planning and delivery, though they still remain under investigation for clinical implementation.

### Synthetic data

AI-generated data is increasingly important in imaging, enabling the creation of synthetic CT (sCT) and MRI (sMRI) that maintain anatomical accuracy. For example, Zhou et al developed a generative adversarial network (GAN) based framework capable of generating both sCT and sMRI from a single T1-weighted MRI for nasopharyngeal patients.^7^ Similarly, Szmul et al employed a cycleGAN to generate sCTs from cone beam CTs (CBCTs) for paediatric abdominal patients,^8^ while Zhu et al also used a cycleGAN to generate high-quality sMRI from low-resolution images, reducing scan times.^9^ Additionally, Tracey et al used synthetic data to train a convolutional neural network (CNN) for automated MRI QA by integrating patient scans with simulated coil malfunctions, demonstrating the utility of synthetic datasets for training robust AI models.^10^

### Image registration

AI has significantly advanced the accuracy and speed of image registration, a critical step in aligning medical images for treatment planning. Two main methods, 2D-3D and 3D-3D registration, play distinct roles in radiotherapy workflows. 2D-3D registration aligns 2D X-ray images to 3D planning CT datasets, aiding in real-time patient setup and treatment verification. Jaganathan et al developed a self-supervised DL framework for rigid registration of X-rays to CT images, improving registration efficiency.^11^ 3D-3D registration involves aligning volumetric datasets, such as CT-to-MRI or CT-to-CT scans, which is essential for multimodality treatment planning and adaptive workflows. Ho et al proposed a lung registration network based on an enhanced U-net architecture to deformably register full-inspiration and end-expiration CT images,^12^ while De Frutos et al utilized a U-net variant to enhance image-to-image registration for abdominal CTs, with additional applications in brain MRIs through transfer learning.^13^

## Organ and target segmentation

AI is integral to autosegmentation in CT and MR imaging, with multiple commercial systems now offering AI-based autocontouring solutions. Siemens Healthineers^14^ and Phillips^15^ provide multimodality software integrated with their scanners, while TheraPanacea^16^ offers web-based tools for autocontouring, image registration, and sCT generation from MR or CBCT images. Beyond these systems, AI is being explored for advanced modalities like PET-MR. For instance, Liedes et al utilized a 2D U-net to autosegment head and neck (H&N) organs at risk (OARs) from fused PET-MR images,^17^ while Shiri et al developed a federated learning network for PET/CT segmentation, enabling multi-institutional learning while preserving patient privacy.^18^

Uncertainty estimation is the next frontier in autosegmentation, crucial for building trust in AI tools. By mapping uncertainty, physicians can validate AI-generated contours and address scepticism around fully automated systems. Maruccio et al employed a 3D-U-net to generate probability and uncertainty maps for autosegmentation of lung cancer gross tumour volumes from CT images,^19^ and Min et al used a 3D-ResUnet with a regression classifier to delineate prostate clinical target volumes on MR images, highlighting areas of uncertainty.^20^ Given the breadth of ongoing research in autocontouring, another article in this edition delves deeper into AI’s role in this field. As such, this paper provides only a high-level overview of its applications and current research.

## Treatment planning

The creation of treatment plans is a critical step in the radiotherapy process, where AI has been explored to predict dose distributions, automate planning workflows, and refine treatment parameters. These advancements aim to precisely target tumours while sparing healthy tissue, with the goal of enhancing safety, efficacy, and personalization. Applications range from research-stage innovations to clinically implemented tools, underscoring AI’s growing impact on radiotherapy.

### Dose distribution prediction

GANs, U-nets, and transformer-based architectures have demonstrated significant advancements in dose distribution prediction. Chandran et al’s MemU-net, a modified U-net model, outperformed traditional U-net and GAN approaches in predicting dose distributions.^21^ Similarly, Shen et al’s double cascade U-net combined with finite element methods highlighted DL’s ability to optimize dose predictions for cervical cancer treatments,^22^ while Hu et al introduced a transformer-based U-shaped network for enhanced feature extraction in H&N dose predictions.^23^ Additionally, Buatti et al demonstrated how AI-generated contours could compensate for incomplete datasets, improving dose prediction accuracy for H&N cases.^24^ Though still under investigation, these models provide a strong foundation for more efficient radiotherapy workflows.

### Automated treatment planning

Automated treatment planning incorporates methodologies like knowledge-based planning (KBP) and DL-based approaches to enhance efficiency and reduce manual workload. KBP uses machine learning (ML) to generate plans from prior treatment data, ensuring consistency across plans. Commercial systems like Varian’s *RapidPlan*^25^ and RaySearch’s *RayStation*^26^ utilize KBP to create consistent, high-quality automated treatment plans. Current research focuses on advancing KBP systems; for example, Huang et al developed an end-to-end volumetric arc therapy (VMAT) planning framework for rectal cancer, integrating DL-based segmentation with *RapidPlan* to fully automate contouring and planning workflows.^27^

In DL-based approaches for dose estimation, models such as U-nets are employed to predict dose distributions directly, eliminating the need for traditional planning methods. These methods involve training models to predict dose distributions from treatment planning images, which can then be used for optimization. For instance, Maes et al used a 3D-U-net within *RayStation* to predict dose distributions for proton pencil beam treatments, which were optimized into deliverable plans for chest wall cases.^28^ While this process reduces the amount of manual work required, these models do not directly predict treatment plans themselves.

Recent innovations bypass dose predictions entirely, offering direct pathways to generating treatment plans. Heilemann et al proposed an encoder-decoder network to directly predict monitor units, leaf positions, and jaw positions for prostate VMAT plans.^29^ Similarly, Hrinivich et al employed deep reinforcement learning to iteratively predict and optimize machine parameters for single-arc VMAT plans.^30^ These advancements aim to streamline workflows and reduce dependence on manual planning.

### Treatment outcome prediction

AI is increasingly used to predict treatment outcomes, such as survival probabilities, side effects, and metastasis risks, providing valuable insights for refining plans. Cui et al developed actuarial DL models to predict radiation pneumonitis and local control in stage III non-small cell lung cancer (NSCLC) patients, enabling clinicians to optimize treatment parameters.^31^ Similarly, Humbert-Vidian et al compared ML models to predict mandible osteoradionecrosis in H&N radiotherapy, leveraging demographic, clinical, and dosimetric variables for case-specific evaluations.^32^ Zhang et al developed random forest models to detect early radiation-induced brain injury in nasopharyngeal carcinoma patients undergoing 3D conformal radiotherapy (3D-CRT), providing a tool for mitigating adverse effects early in the treatment process.^33^ These tools show potential in improving patient care, though further validation is necessary before broad clinical adoption.

### Dose optimization

AI-driven dose optimization refines plans to balance tumour coverage with healthy tissue preservation. Unlike outcome prediction models, dose optimization focuses on enhancing radiation delivery precision. For instance, Maragno et al used optimization-constrained learning to improve NSCLC treatment plans by predicting potential radiation-induced toxicities.^34^ Similarly, Dudas et al employed a survival-NN to identify dosimetric criteria for stereotactic body radiation therapy (SBRT), reducing recurrence risks while maintaining effective dose levels.^35^ Sun et al integrated expert knowledge with AI recommendations to personalize dose prescriptions for NSCLC patients, fostering a collaborative approach to planning.^36^ Although these methods remain in the research phase, they show significant potential for improving radiotherapy outcomes with further validation.

## Quality assurance

The next step in the radiotherapy process is QA, which ensures accurate and safe treatment delivery after plan creation. AI has demonstrated significant potential in enhancing QA processes through patient-specific QA (PSQA), machine-specific QA, and workflow integration.

### Patient-specific QA

AI tools like CNNs, supervised learning models, and other ML frameworks have shown promise in improving PSQA by predicting gamma passing rates and identifying potential errors in treatment plans. By flagging problematic plans before physical measurements are initiated, these methods reduce QA workloads and improve efficiency while maintaining accuracy. Noblet et al investigated nine ML frameworks to predict PSQA outcomes, integrating them with Eclipse TPS for efficient QA predictions.^37^ Pillai et al used a voting classifier composed of five ML models to flag challenging PSQA cases requiring more detailed reviews by physicists,^38^ while Salari et al employed supervised learning models to predict gamma passing rates in VMAT plans, enabling error identification before physical measurements.^39^ Liu et al demonstrated that CNNs could outperform traditional gamma pass rate methods by detecting dose, gantry, collimator, and couch errors in IMRT and VMAT plans across various sites.^40^ Though validation is ongoing, these advancements highlight AI’s potential to enhance PSQA efficiency.

### Machine-specific QA

AI tools for machine-specific QA, such as regression models and neural representation methods, are being explored to streamline routine checks for linear accelerators (linacs). By reducing the effort required for data collection and analysis, these approaches improve efficiency without compromising machine performance. Zhao et al used a multivariate regression model to predict beam percent depth doses and profiles from a single measurement, reducing the need for extensive scans.^41^ Liu et al employed a neural representation model with a multilayer perceptrons to model beams from sparse measurements, offering a more efficient approach to routine QA.^42^ Additionally, advanced DL combinations are being developed for predictive maintenance, offering proactive solutions for addressing equipment issues before they arise. Petchuchart et al combined a CNN and long-term short memory framework to predict daily trends and next-day records for ring gantry linacs, enabling predictive maintenance and proactive resolution of equipment issues.^43^ Though still in development, these approaches demonstrate AI’s potential to enhance routine QA and improve efficiency in radiotherapy workflows.

### Integration of AI in workflows

Beyond QA-specific tasks, AI offers opportunities to streamline operational workflows, including optimization of patient scheduling and physics tasks. Petragallo et al showed that automating portions of weekly chart checks improved time management and accuracy for clinical physicists.^44^ Neylon et al used CNNs to automate image reviews during chart checks, flagging patients who might require further evaluation.^45^ AI has also been applied to appointment scheduling in radiotherapy clinics, aiming to reduce waiting times and optimize resource use. Pham et al proposed a ML algorithm that reduced waiting times for palliative patients while maintaining consistent scheduling for curative treatments.^46^ Similarly, Xie et al developed a NN to predict treatment times per fraction for dynamically scheduling appointments, achieving a 10% improvement in linac utilization.^47^ These advancements highlight AI’s potential to create systems integrating QA, scheduling, and workflows. By enhancing communication across tasks, AI can transform radiotherapy into a cohesive, efficient process that improves safety and patient care.

## Patient identification and setup

Accurate patient identification and setup are critical to ensuring safe and effective treatments. AI-based tools are being investigated to address challenges such as misidentification and alignment errors, offering the potential to improve safety, precision, and efficiency during treatment processes.

### Identification and safety

Traditional processes for verifying patient identity, such as manual checks or visual confirmation, can be error-prone and increase the risk of misidentification. To address these challenges, AI tools are being developed to automate and enhance patient identification. Rajurkar et al utilized OpenCV and a Haar Cascade classifier to create a *Patient Identification System*, which uses a camera to identify faces within a 1-meter field of view on the maze wall.^48^ For future clinical use, the authors also propose the integration of this system with treatment consoles to include lock-out-features if the patient is not positively identified.

### Patient setup

Conventional patient setup methods often rely on manual CBCT alignment or basic auto-registration algorithms, which depend heavily on human interpretation and can struggle with complex cases. AI applications are being studied to enhance the precision of patient setup by detecting and reducing alignment errors. For instance, Luximon et al developed a CNN-based error detection model that identifies vertebral body misalignments by analysing CBCT and simulation CT images, achieving a mean absolute error (MAE) of 1.7 mm across multiple sites.^49^ Similarly, Petragallo et al utilized a CNN for detecting alignment errors between kV images and digitally reconstructed radiographs, reducing the MAE for vertebral body alignment from 2.3 mm for manual methods to 0.6 mm using the AI model.^50^ Neylon et al expanded this model to detect pre-treatment misalignment shifts for H&N and pelvic patients, further improving setup precision across various sites.^45^ These AI-driven advancements enable more precise and efficient positioning of patients during treatments, reducing setup errors and improving accuracy, although further validation is required for clinical implementation.

## Treatment and treatment delivery

Artificial intelligence has shown significant potential in enhancing treatments by improving delivery techniques, optimizing advanced treatment modalities, and enabling personalized approaches such as ART. While conventional techniques such as 3D-CRT, IMRT, and VMAT have primarily seen AI integration earlier in the workflow, as discussed in previous sections. In contrast, newer research targets AI applications such in delivery optimization and in advanced modalities such as brachytherapy, proton therapy, and stereotactic treatments. These developments aim to enhance precision, personalize treatments, and improve patient outcomes. While promising, most of these approaches remain under investigation and require further clinical validation.

### Delivery techniques

AI-based tools are advancing delivery techniques, particularly in gating and motion management. These techniques adapt treatments to patient movements, such as respiratory motion, and can enhance precision in MRgRT. Shi et al developed a DL model to track respiratory motion during treatment, enabling motion-adaptive therapy with enhanced accuracy.^51^ Jeong et al compared AI models to predict patient stress levels during treatments and their impact on respiratory patterns, improving motion management for lung treatments.^52^ For MRgRT, Hunt et al used VoxelMorph, a deformable image registration algorithm, to track motion in real time, enhancing treatment precision.^53^ Additionally, Lombardo et al reviewed current AI-based motion management techniques for MRgRT and proposed a workflow for real-time motion management, offering a roadmap for clinical integration.^54^

### Advanced treatment modalities

Advanced treatment modalities such as brachytherapy, proton therapy, and stereotactic techniques present unique challenges and opportunities where AI is being leveraged to enhance precision, improve safety, and streamline clinical workflows.

#### Brachytherapy

In brachytherapy, AI has been employed to streamline processes like autosegmentation, dose prediction, and adaptive planning. Hampole et al developed a CNN for autosegmentation of the prostate clinical target volume (CTV) from trans-rectal ultrasound images, improving accuracy and reducing manual workload.^55^ Xie et al designed a CNN to localize interstitial needles from CT images in cervical brachytherapy, facilitating efficient planning.^56^ For dose prediction, Quetin et al applied U-net models to generate high-resolution dose maps in high dose rate breast brachytherapy,^57^ while Berumen et al used a 3D-U-net to calculate dose-to-medium-in-medium distributions for low dose rate prostate brachytherapy.^58^

#### Proton therapy

To support the unique delivery requirements of proton therapy, recent innovations in AI have focused on enhancing range and dose verification during treatment. Lerendegui-Marco et al developed a ML-supported prompt gamma imaging system using a Compton camera-based detector (i-TED), improving signal-to-background separation and enabling real-time range verification.^59^ Yabe et al proposed a DL method for in vivo dose verification using secondary-electron bremsstrahlung imaging, demonstrating strong performance under low signal conditions.^60^ Together, these studies highlight AI’s direct integration into treatment delivery can improve range accuracy and dose precision in proton therapy.

#### SBRT and SRS

AI has also been investigated in stereotactic body radiation therapy (SBRT) and stereotactic radiosurgery (SRS) to improve dose prediction and treatment response modelling. Li et al used a cascaded-deep-supervised CNN to predict dose distributions in gamma knife radiosurgery, enhancing planning precision.^61^ Pan et al compared two 3D-U-net architectures for dose prediction in fixed-field IMRT-based SRS, improving plan quality for brain metastases,^62^ while Brodin et al employed a gradient-boosted regression tree algorithm for dose prediction of OARs in lung SBRT, enhancing patient safety through better dose optimization.^63^

### Adaptive radiotherapy

ART represents one of the most promising applications of AI in treatment delivery, enabling real-time personalization across fractions. Models are being developed to rapidly adjust treatment plans in response to anatomical changes, improving precision while reducing manual planning burden. Hooshangnejad et al developed *deepPERFECT*, a GAN designed to accelerate adaptive workflows for NSCLC patients, reducing planning time from 4 to 2 weeks.^64^ Niraula et al introduced *ARCLiDS*, a clinical decision support system that estimates treatment outcomes and provides recommendations for dose adjustments during ART.^65^ In proton therapy, Zhang et al developed a DL model to generate daily adaptive dose distributions for pencil beam proton therapy, enabling real-time dose personalization based on the patient anatomy.^66^ These advancements demonstrate AI’s ability to enhance the efficiency and personalization of ART, offering a significant step forward in precision medicine. Looking ahead, future advancements may explore biological personalization, where treatments are tailored to tumour-specific features such as hypoxia levels or radiosensitivity to further optimize outcomes.

## Guidelines and regulations

As AI continues to expand in medicine, regulatory bodies are recognizing the need for frameworks to ensure its safe and ethical integration into clinical practice. Federal regulations address transparency and reliability, while radiotherapy-specific professional guidelines focus on validating and reporting AI tools for clinical practice.

### Current regulations

In March 2024, the FDA published a guide outlining key principles for AI development in medical products.^67^ These include requirements for transparency, robustness, and the use of real-world evidence to support AI models. However, the United States has not enacted federal regulations specific to AI in healthcare, leading to variability in adoption and oversight as individual states implement their own rules. For instance, Colorado’s AI Act^68^ (May 2024) regulates patient data privacy, while Illinois’ AI Systems Act^69^ (Aug 2024) mandates oversight of AI-driven healthcare coverage decisions.

On a global scale, the EU AI Act, also enacted in 2024, classifies AI systems used in medical devices as “high-risk”.^70^ This classification imposes stricter requirements for validation, reporting, and post-market surveillance. For radiotherapy, this means that tools such as dose prediction models or adaptive planning systems must undergo rigorous testing to ensure safety and efficacy, potentially delaying their adoption in clinical practice. However, these regulations also set a precedent for accountability, offering a blueprint for other nations.

### Professional guidelines

In radiotherapy, professional organizations are also taking steps to guide the responsible integration of AI. The AAPM and European Society for Radiotherapy and Oncology (ESTRO) joint guideline, published in June 2024, provides a comprehensive framework for AI in radiotherapy.^71^ Key recommendations emphasize model availability, transparency, and standardized reporting to ensure reproducibility for clinical integration.

### Ethical considerations

Despite these advancements, ethical challenges remain, including algorithmic bias, patient data privacy, and equitable access to AI tools. Bias in algorithms can lead to unequal treatment recommendations, disproportionately affecting underserved populations.^72^ Reliance on proprietary datasets risks limiting inclusivity if they fail to reflect diverse populations.^73^ Addressing these issues and ensuring clinicians are trained to use AI responsibly is critical for safe and equitable integration into healthcare.

### Future directions

Looking ahead, there is a growing need for global harmonization of AI regulations. Establishing interdisciplinary collaborations between regulatory bodies, clinicians, and software developers could accelerate the safe adoption of AI in radiotherapy. These efforts will help bridge gaps between innovation and implementation, ensuring that AI improves patient care while maintaining high safety and ethical standards.

## Clinical trials

AI tools are increasingly being used to process and analyse the vast amounts of data generated in clinical trials, offering capabilities such as identifying flawed data, predicting outcomes, and analysing treatment efficacy. Companies like Clario have developed AI-integrated platforms that facilitate access to large databases, streamline data analysis, and improve trial efficiency.^74^

### Guidelines for integration

To guide the integration and reporting of AI in clinical trials, specific guidelines have been established to ensure consistency, transparency, and ethical oversight. SPIRIT-AI details recommendations for designing trials that incorporate AI in interventional measures,^75^ while CONSORT-AI outlines standards for reporting AI usage in trials, focusing on reproducibility and minimizing bias in reported outcomes.^76^ These frameworks are essential for ensuring that AI-driven insights are both reliable and actionable.

### AI in radiotherapy-focused trials

AI is being explored in various radiotherapy-focused clinical trials listed on ClinicalTrials.gov,^77^ showcasing its potential across observational and interventional studies. Observational trials include studies on radiomics and AI-assisted tumour/OAR segmentation, demonstrating how AI can extract meaningful patterns from imaging datasets. Interventional trials focus on ART and AI-driven dose recommendations for lung SBRT, highlighting AI’s ability to inform real-time treatment adjustments and enhance personalization.

### Future directions and challenges

Future direction for AI in clinical trials includes leveraging large trial datasets to train models that can predict outcomes earlier, identify trends, and refine trial designs in real time. These advancements could accelerate trial efficiency and patient classification. However, ensuring robust oversight, as outlined above for regulations, remains essential. Transparency, reproducibility, and collaboration will be critical to balance innovation with ethical and equitable implementation in clinical trials.

## Conclusion

Artificial intelligence is revolutionizing radiotherapy by addressing longstanding challenges and enhancing every step of the treatment process. From improving dose prediction and treatment planning to optimizing delivery and QA, AI is driving precision and personalization in patient care. Innovations like ART and real-time motion tracking demonstrate its potential to create safer, more efficient workflows and advance the future of cancer treatment.

Despite AI’s potential, many tools remain in development and need further validation before clinical integration. Regulatory frameworks, like those from the FDA, EU, AAPM, and ESTRO, offer essential guidance for safe, ethical implementation. Addressing challenges like algorithmic bias, data diversity, and the lack of standardized methodologies will be critical to ensuring equitable and reliable outcomes.

As AI accelerates clinical trials and innovative treatments, its integration into radiotherapy will require collaboration among clinicians, researchers, and policymakers to bridge the gap between innovation and application. AI has the potential to redefine radiotherapy by building on current advancements, fostering interdisciplinary partnerships, and addressing limitations. This will enable safer, more effective, and personalized treatments that improve patient outcomes worldwide.

## Statement to the editor

This review was invited by the journal for submission.
